# The -174G/C Interleukin-6 Gene Promoter Polymorphism as a Genetic Marker of Differences in Therapeutic Response to Methotrexate and Leflunomide in Rheumatoid Arthritis

**DOI:** 10.1155/2016/4193538

**Published:** 2016-09-21

**Authors:** A. J. Ruiz-Padilla, J. I. Gamez-Nava, A. M. Saldaña-Cruz, J. D. Murillo-Vazquez, M. L. Vazquez-Villegas, S. A. Zavaleta-Muñiz, B. T. Martín-Márquez, J. M. Ponce-Guarneros, N. A. Rodriguez Jimenez, A. Flores-Chavez, F. Sandoval-Garcia, J. C. Vasquez-Jimenez, E. G. Cardona-Muñoz, S. E. Totsuka-Sutto, L. Gonzalez-Lopez

**Affiliations:** ^1^Departamento de Medicina Interna-Reumatología, Hospital General Regional 110, IMSS, 44710 Guadalajara, JAL, Mexico; ^2^Doctorado en Farmacología, Centro Universitario de Ciencias de la Salud (CUCS), Universidad de Guadalajara (U de G), 44340 Guadalajara, JAL, Mexico; ^3^Centro Universitario de Ciencias de la Salud, Universidad de Guadalajara, 44340 Guadalajara, JAL, Mexico; ^4^Unidad de Investigación en Epidemiología Clínica, Centro Médico Nacional de Occidente (CMNO), Instituto Mexicano del Seguro Social (IMSS), Hospital de Especialidades, 44340 Guadalajara, JAL, Mexico; ^5^Centro Universitario de Investigaciones Biomédicas (CUIB), Universidad de Colima, 28040 Colima, COL, Mexico; ^6^Departamento de Epidemiología, Unidad Médica Familiar 4, IMSS, 44220 Guadalajara, JAL, Mexico; ^7^Departamento de Salud Pública, CUCS, U de G, 44340 Guadalajara, JAL, Mexico; ^8^División de Posgrado, Facultad de Ciencias de la Salud, Universidad Juárez del Estado de Durango, 35050 Gómez Palacio, DGO, Mexico; ^9^Instituto de Investigación en Reumatología y del Sistema Músculo Esquelético (IIRSME), CUCS, U de G, 44340 Guadalajara, JAL, Mexico; ^10^Unidad Médica Familiar 97, IMSS, 46470 Magdalena, JAL, Mexico; ^11^Departamento de Fisiología, CUCS, U de G, 44340 Guadalajara, JAL, Mexico

## Abstract

*Objective*. To evaluate the association of -174G/C* IL-6* polymorphism with failure in therapeutic response to methotrexate (MTX) or leflunomide (LEF). This prospective, observational cohort included 96 Mexican-Mestizo patients with moderate or severe rheumatoid arthritis (RA), initiating MTX or LEF, genotyped for* IL-6* -174G/C polymorphism by PCR-RFLP. Therapeutic response was strictly defined: only if patients achieved remission or low disease activity (DAS-28 < 3.2).* Results*. Patients with MTX or LEF had significant decrement in DAS-28 (*p* < 0.001); nevertheless, only 14% and 12.5% achieved DAS-28 < 3.2 at 3 and 6 months. After 6 months with any of these drugs the -174G/G genotype carriers (56%) had higher risk of therapeutic failure compared with GC (RR: 1.19, 95% CI: 1.07–1.56). By analyzing each drug separately, after 6 months with LEF, GG genotype confers higher risk of therapeutic failure than GC (RR = 1.56; 95% CI = 1.05–2.3; *p* = 0.003), or CC (RR = 1.83; 95% CI = 1.07–3.14; *p* = 0.001). This risk was also observed in the dominant model (RR = 1.33; 95% CI = 1.03–1.72; *p* = 0.02). Instead, in patients receiving MTX no genotype was predictor of therapeutic failure. We concluded that* IL-6* -174G/G genotype confers higher risk of failure in therapeutic response to LEF in Mexicans and if confirmed in other populations this can be used as promissory genetic marker to differentiate risk of therapeutic failure to LEF.

## 1. Introduction

Rheumatoid arthritis (RA) is a chronic, systemic, inflammatory disease that involves synovial joints and other organs and it is associated with impairment in physical function, higher morbidity, and premature mortality [1]. Multiple guidelines of treatment for RA recommend, as first-line treatment, the use of conventional synthetic disease-modifying antirheumatic drugs (cs-DMARDs), with methotrexate (MTX), as the cornerstone of the majority of the therapeutic schemes [2–4]. Leflunomide (LEF) was the last cs-DMARD to appear before the biologic-DMARDs era, and LEF is considered an alternative as first-line treatment in patients with RA with intolerance to or contraindication for MTX [4]. In developing countries with serious economic limitations for the utilization of biologic-DMARDs, treatment based on MTX or LEF constitutes an alternative, frequently employed as monotherapy or combined therapy. In Mexico, a multicenter study performed, with the aim of describing the drugs most frequently utilized for the treatment of RA and ankylosing spondylitis, found that around 72.3% of 1,096 patients with RA were taking MTX and 18.5% LEF [5]. However, a significant number of patients are considered nonresponders to cs-DMARDs. Several works have reported wide variability in the efficacy of MTX or LEF. Strand et al. reported that the rate of response in ACR20 for MTX is only 46% and for LEF 52%, but only 20% with LEF and 9% with MTX achieved therapeutic response in ACR70 [6]. In fact, other authors observed a higher response rate using ACR20 criteria: 62% of responders to LEF and 54% to MTX [7]. According to treat-to-target concepts, one of the main objectives in the treatment of RA is to maintain remission of the disease activity or at least the achievement of low disease activity [8]. Unfortunately, a high proportion of patients with monotherapy with cs-DMARDs do not achieve these therapeutic targets. A concern regarding the treatment of RA comprises earlier recognition of patients with factors that predict a lack of efficacy in order to modify the therapeutic strategy. Cytokines constitute important mediators of the immune and inflammatory response and play an important role in the pathophysiology of joint inflammation and destruction in RA [9]. Among these cytokines, interleukin-6 (IL-6) has a relevant role in the perpetuation of synovial joint inflammation in RA, being widely related with disease activity [10, 11] and radiologic joint damage [12]. Some authors have described that genetic differences in the expression of IL-6 can be related with more severe disease [13]. The -174G/C IL-6 gene promoter polymorphism (rs1800795), localized in the negative regulative domain of the IL-6 gene promoter, is involved in transcriptional regulation [14, 15], whereas Konenkov et al. identified that GG genotype was associated with higher IL-6 serum levels [16].

Several studies have suggested that the -174G/C* IL-6* polymorphism may constitute a genetic marker for identifying a predisposition for therapeutic response to biologic-DMARDs [17–19]. However, only a single group of authors, to our knowledge, have examined, in two separate studies, the influence of the -174G/C* IL-6* gene promoter polymorphism on therapeutic response to MTX or LEF [20, 21]. Pawlik et al. analyzing -174G/C* IL-6* gene promoter polymorphism in RA patients identified that genotype GG may confer a risk for lower response to MTX compared with genotypes GC and CC [20]. Instead, the -174G/C polymorphism may not affect therapy outcomes in patients with RA treated with LEF [21]. Although these studies presented interesting findings, two major limitations can be observed. First, these two studies investigate therapeutic response using ACR20 or ACR50 as the main outcome measures; these are well-validated scales for clinical trials, but not for cohort studies, and neither ACR20 nor ACR50 provide sufficient information of disease severity at baseline. Second, because these two studies analyzed the effects of MTX and LEF in the presence of the -174G/C* IL-6* gene promoter polymorphism separately, they were unable to evaluate the overall response to both of these cs-DMARDs and to compare if -174G/C* IL-6* gene promoter polymorphism is predictor of the therapeutic response comparing both drugs. A more strict definition of therapeutic response should be applied to achieve the aim of maintaining remission or at least low disease activity with the therapy; in this regard, it is highly recommended to use DAS-28 < 3.2 as main outcome measure. Therefore, the aim of this observational cohort study was to evaluate the association of -174G/C* IL-6* polymorphism with failure in therapeutic response to MTX or LEF in Mexican-Mestizo women with RA.

## 2. Materials and Methods

### 2.1. Study Design

This study was a prospective, observational cohort (patients were included in a period covering October 2014 to December 2015) of patients with established moderate or severely active RA who initiated MTX or LEF for the treatment of their disease.

### 2.2. Clinical Setting

This was a single-center study performed at an outpatient rheumatology clinic of a secondary-care hospital in Guadalajara, Mexico (Hospital General Regional 110, IMSS). These patients were referred by primary-care physicians from a primary-care clinic.

### 2.3. Patients

Inclusion criteria comprised patients with RA according to American College of Rheumatology (ACR) 1987 criteria [22], >18 years of age, with an active disease defined as a Disease-Activity Score (DAS-28) index of 28 joints with a score of >3.2, with Mexican-Mestizo ethnicity (defined as individuals who, for three generations including their own, were born in Mexico and who were descendants of the original autochthonous inhabitants of the region and of individuals who were mainly Spaniards) [23], and all of these with at least 6 months or more without MTX or LEF. In the case of patients with a familial history of RA, only one case by family was included. Patients were excluded if they had antecedents of or concomitant therapy with biologic-DMARD; also, we excluded patients with chronic infections including hepatitis B or C infections, human immunodeficiency virus (HIV) infections, overlapping syndrome with other rheumatic diseases such as Systemic Lupus Erythematosus (SLE), or patients who had an increase of transaminases of >2-fold of normal values, pulmonary fibrosis, serum creatinine > 1.2, or any other contraindications for MTX or LEF or had a ≤3.2 DAS-28 score. A total of 177 patients were screened for the study; among these, 81 were excluded due to the following reasons: two patients presented a <3.2 DAS-28 score; there were 48 patients with previous history of MTX or LEF suspended because of toxicity or noncompliance, and 31 patients did not accept to be included in the 6-month follow-up with the same drug.

At the time of inclusion in the study, all patients were initiating MTX or LEF as therapy for disease control in RA and had moderate or severe disease activity defined as a score of >3.2, according to the validated modified Disease Activity Score (DAS-28) index for 28 joints [24].

### 2.4. Baseline Evaluation

*(a) Clinical Evaluation*. All patients were interviewed to assess clinical and sociodemographic characteristics. Patients were evaluated regarding their functioning, using the validated version for Mexicans of the Health Assessment Questionnaire-Disability Index (HAQ-Di) [25]. Additionally, DAS-28 was evaluated by trained evaluators and a history of RA medication was obtained. Rheumatoid Factor (RF) in IU/mL (Dade Behring, DE, USA) was quantified in serum by nephelometry. Positive RF was considered >20 IU/mL. Erythrocyte Sedimentation Rate (ESR) was determined employing the Wintrobe technique.

*(b) Determination of Serum IL-6 Levels*. These were measured with an Enzyme-Linked Immunosorbent Assay (ELISA) utilizing commercial kits (R&D Systems, Minneapolis, MN, USA). This kit has a detection range from 3.10 to 300 pg/mL, and the Minimal Detectable Dose (MDD) of IL-6 is <0.70 pg/mL.

*(c) DNA Isolation and Genotyping.* Genomic DNA was obtained by the Miller method [26] from the patients' peripheral blood that was collected in tubes containing EDTA. Genotype was screened by an approach based on Polymerase Chain Reaction-Restriction Fragment-Length Polymorphism (PCR–RFLP), and* SfaNI* restriction endonuclease was used, as described elsewhere [27]. The resulting fragments were analyzed by electrophoresis in a 6% polyacrylamide gel stained with silver nitrate. The resulting genotypes for both polymorphisms were classified in one of the following three categories: nonexcisable homozygote genotype (CC); excisable homozygote (GG), and heterozygote (CG). All sample genotyping was carried out by three researchers blinded to the clinical characteristics and evolution of the patients included in the test, which included quality-control samples with experimental samples for validation.

### 2.5. Follow-Up of the Cohort

All patients with RA initiating MTX or LEF were evaluated by three researchers trained in the clinical parameters of RA at the baseline and at 3 and 6 months. Differences in the DAS-28 index at 3 and 6 months regarding baseline values were obtained. We classified all patients according to therapeutic response, which was defined according to treat-to-target guidelines, as patients that have reached at least low disease activity or remission of RA. Operatively, these patients in order to be classified as responders have undergone the treatment DAS-28 ≤ 3.2.

### 2.6. Statistical Analysis

A comparison between these two groups at baseline was performed using the unpaired Student's *t*-test in order to compare differences in means, and chi-square test (or Fisher exact test) was utilized to compare differences in proportions between groups. Relative Risks (RR) for therapeutic response and their 95% Confidence Intervals (95% CI) were obtained at 3 and 6 months.

Allele and genotype frequencies of both polymorphisms were obtained by direct counting. Genotype and allele frequencies were compared using the chi-square test (or the Fisher exact test if required). We initially examine failure rates for therapeutic response for each genotype of the -174G/C* IL-6* polymorphism separately at 3 or 6 months. Thereafter, we performed the RR analysis in three forms as follows: (a) rate of therapeutic failure in patients with GG genotype divided by rate of therapeutic failure in patients with GC and CC genotypes separately; (b) after that, analyzing the risk of therapeutic failure in patients with GG genotype versus rate of therapeutic failure in patients with GG or GC genotypes (dominant model); and (c) finally, examining RR for therapeutic failure employing the rate of failure to therapy in patients with GG or GC genotype divided by rate of therapeutic failure in patients with CC genotype (recessive model) of the -174G/C polymorphism of the* IL-6* gene. A similar approach was utilized for a subanalysis of patients treated with MTX or LEF separately. The *p* value was set at 0.05 level. All of the statistical analyses were performed using the software SPSS software 20.0 (SPSS Inc., Chicago, IL).

## 3. Results

Table 1 describes the baseline characteristics of 96 patients included in the cohort. Mean age of these patients was 50.6 years and 97% were females. Patients had mean disease duration of 7.6 years, a HAQ-Di score of 0.95, a DAS-28 of 5.6, the mean titles of RF of 83.2 IU/mL, and a mean glucocorticoid dose of 2.0 mg. The cs-DMARDs used by the patients during the study were the following: 57.3% received MTX and 42.7% received LEF. All patients were genotyped for the presence of -174G/C* IL-6* promoter polymorphisms, and the following genotype frequencies were observed: GG (56%), GC (32%), and CC (12%). Low rate of response using the strict criteria of achieving low disease activity or remission was achieved in the total group independently of the drug: response rates were 14% at 3 months and 12.5% at 6 months. There was a rate of response of 12.7% at 3 and 6 months in patients receiving MTX and of 17% at 3 months and of 12% at 6 months. No statistical differences were observed in the rate of responders between LEF and MTX at 3 or 6 months.

Comparison of the clinical and genetic characteristics between patients with MTX versus LEF demonstrated a difference in disease duration (6.05 years, MTX, versus 9.2 years, LEF; *p* = 0.05). Other variables evaluated did not achieve statistically significant differences. In data that are not shown in tables the response rates for MTX were 12.7% at 3 months and the same percentage at 6 months, while those of LEF were 17.1% at 3 months and 12.2% at 6 months.

In data not shown in the tables, we observed a significant decrease in the DAS-28 index score employed as the quantitative variable at 3 or 6 months independently of treatment with MTX or with LEF. At cohort onset, the DAS-28 index score was 5.6 ± 1.1 and, on being evaluated at 3 months, decreased to 4.56 ± 1.12 (*p* < 0.001), with a similar decrease at 6 months with results of 4.49 ± 0.08 (*p* < 0.001). In a separate analysis, patients with MTX obtained a DAS-28 score at baseline of 5.65 ± 1.10; at 3 months, this decreased to 4.68 ± 1.16 (*p* < 0.001) and at 6 months, to 4.44 ± 1.06 (*p* < 0.001). LEF obtained for DAS-28 at baseline was 5.57 ± 1.13; at 3 months, this decreased to 4.39 ± 1.05 (*p* < 0.001) and at 6 months, to 4.57 ± 1.12 (*p* < 0.001).

Table 2 compares sociodemographic and clinical characteristics between patients who are GG genotype carriers and patients with GC or CC genotype. GG genotype carriers exhibited significantly higher RF levels (*p* = 0.009) and ESR (*p* = 0.02) compared with GC or CC genotype. No other variables achieved statistical significance, although there was a nonsignificant trend to higher IL-6 levels in GG genotype carriers.

Table 3 shows therapeutic failure in patients with RA with MTX or LEF during follow-up at 3 or 6 months according to the -174G/C* IL-6* gene polymorphism. After 3 months of treatment, a higher percentage of GG genotype was observed in nonresponders (87.0%) as well as at 6 months (92.6%); in the comparison between genotypes associated with nonresponse at 6 months of follow-up, GG compared with GC confers significantly more risk for therapeutic failure (RR = 1.19; 95% CI = 1.07–1.56; *p* = 0.03). The remaining comparisons of genotype or allele evaluated did not achieve statistically significant differences.

In Table 4, the analysis shows the therapeutic failure in patients with MTX during follow-up at 3 or 6 months according to the -174G/C* IL-6* gene polymorphism. The number of patients carrying the GG genotype was slightly increased in the group of nonresponders compared with responders without statistical significance. After 3 months of treatment, the GG genotype was observed in 90.9% of nonresponders and at 6 months in 87.9% of the same group. All the comparisons between rates of response according to genotype or allele did not achieve statistically significant differences.

Table 5 presents therapeutic failure in patients with RA treated with LEF during follow-up at 3 or 6 months according to the -174G/C* IL-6* gene polymorphism. There were no statistical differences after 3 months of treatment; however, at 6 months, the GG genotype carriers had a higher risk of therapeutic failure compared with GC genotype carriers (RR = 1.56; 95% CI = 1.05–2.30; *p* = 0.003) and compared with the CC genotype carriers (RR = 1.83; 95% CI = 1.07–3.14; *p* = 0.001); in addition, dominant model GG versus GC + CC increased the risk of nonresponse (RR = 1.33; 95% CI = 1.03–1.72; *p* = 0.02).

## 4. Discussion 

In the overall analysis that included all patients entering into the cohort independently and if they were treated with MTX or LEF, we did not observe that the -174G/C* IL-6* gene polymorphism may confer higher risk for therapeutic failure except in the GG genotype versus GC genotype carriers at 6 months. Nevertheless, when the rates of therapeutic failure of these drugs are analyzed separately we observed that in patients treated with LEF at 6 months of follow-up, GG genotype confers more risk for failure in therapeutic response compared with patients carrying GC or CC genotypes; this higher risk for failure to respond to LEF was also observed in patients using the dominant model (GG versus GC or CC). Instead, by analyzing only patients treated exclusively with MTX, the -174G/C* IL-6* gene polymorphism may not confer differences in the rate of therapeutic failure at 3 or 6 months.

Our results reflect that the -174G/C* IL-6* gene promoter polymorphism has a low influence on the therapeutic failure in patients treated with the two main cs-DMARDs employed currently in RA. For patients treated with MTX, we observed that GG genotype or G allele does not confer a clinically relevant risk for development of therapeutic failure. These results are in disagreement with the results obtained by Pawlik et al., who described a lower remission rate in patients with GG genotype when these patients are compared with carriers of GC and CC genotype [28]. This discordance can be explained by a series of potential confounders that were not evaluated by Pawlik et al. in their study. First, it is relevant that we included patients with moderate or severe disease activity according to the DAS-28 index score instead of only patients with any level of active disease. Patients with moderate or severe disease activity have an expected lower rate of achieving low disease activity or remission compared with patients with lower scores of this index. Second, we used a stricter definition for therapeutic response that is in concordance with the current concepts that show that a major target in the therapy of RA is maintaining remission or at least low disease activity.

Contrary to our observations of noninfluence of the -174G/C* IL-6* gene polymorphism in response to MTX, we observed in patients treated with LEF that the* IL-6* -174G/C polymorphism may confer differences in the therapeutic response to this drug. We observed that GG genotype carriers had a significant higher risk for failure in therapeutic response in patients treated with LEF at 6 months. These data are in disagreement with the results observed by Pawlik et al., who did not observe an association of -174G/C polymorphisms of the* IL-6* gene with therapeutic outcomes in their patients with RA treated with LEF [21]. The reasons for these differences include that many possible confounders can affect the risk of failure in therapeutic response, including differences in the definition of therapeutic failure (we described above that we used a stricter definition compared with that used by Pawlik et al.), other variables such as disease duration, functioning, or the baseline IL-6 levels that were not evaluated. However, some other variables at the baseline are comparable with the Pawlik cohort and our cohort with LEF [21]. Our patients had a mean age of 52.8 years, very similar to the mean age of 52.9 years in Pawlik's cohort [21]. Similarly, the baseline values for DAS-28 were comparable with these two cohorts (DAS-28 of 5.5 in our group with LEF versus 5.3 in the patients included by Pawlik et al.). Instead, our patients with LEF had long-disease duration of RA of 9.2, whereas Pawlik et al. did not present the disease duration of their patients [21]. This long-disease duration may influence the low rate of therapeutic response; instead, patients with short disease duration (early RA) achieve higher rates of therapeutic response when they are treated early with cs-DMARDs [29]. However, because this was an observational study, these patients were treated late with cs-DMARD; therefore, the rate of patients that we expected to achieve low disease activity or remission is lower than that expected by studies employing these drugs earlier. In contrast with our data, Pawlik et al. also did not describe other characteristics in addition to those of disease activity that may contribute to therapeutic response to cs-DMARDs, such as positivity for RF and impairment in functioning assessed by the HAQ-Di [21]. Therefore, it is likely that major confounders may hide in Pawlik's study on the influence of the -174G/C* IL-6* gene polymorphism on failure in therapeutic response. Additionally, at this time, the DAS-28 index constitutes a standard for defining patients who achieve low disease activity or remission; consequently, we used this index to define strictly therapeutic failure and we choose not to use ACR20 or ACR50 indices as in Pawlik's study [21].

It is interesting that although patients treated with MTX or LEF exhibited a significant decrease in mean DAS28 index at 3 and 6 months with respect to baseline values, only a very low proportion of patients achieved the therapeutic target of maintaining low disease activity or remission. This is in accordance with a number of studies pointing out that MTX or LEF used as monotherapy may achieve a low proportion of patients in remission [30, 31].

Patients carrying GG genotype demonstrate higher levels of ESR and RF compared with patients with the GC or CC genotype. A previous study reported that patients who were carriers of the GG genotype had a significantly increased ESR rate; these findings are consistent with our results [32]; however, our results on increased titres of RF are different from those of other authors such as Pavkova Goldbergova, who found that patients who were carriers of the GC genotype had higher levels of RF without significance [33], although these findings are inconsistent with some studies suggesting that the C allele of the -174 polymorphism of* IL-6* is associated with increased levels of IL-6 in general population [34, 35]. This finding was also observed in patients with RA [36], whereas other authors have found that higher IL-6 levels are observed in patients with GG genotype or G allele [16]. Similarly, in our study, we found a trend for higher levels of IL-6 in patients with GG genotype compared with genotype GC or CC, because some of the patients included in our study had been previously treated prior to study entry with other cs-DMARDs such as sulfasalazine, chloroquine, or azathioprine, although these patients suspended these cs-DMARDs at least 3 month before the study entry. Nevertheless, a possible delayed effect of the previous cs-DMARDs contributing to a decrement in serum IL-6 levels in these patients with RA cannot be completely excluded [37]. Therefore, these results about the lack of statistically significant relationship between GG genotype and IL-6 serum levels should be interpreted with caution and studies with patients naïve to cs-DMARDs should be made in the future.

There are several limitations in the present study. First, we were only able to assess the short-time response to these two cs-DMARDs in terms of disease activity at 3 and 6 months; however, we were unable to evaluate the influence of the -174G/C polymorphism of the* IL-6* gene on other relevant responses different from disease activity that develop in the long term and are also determinant of the prognosis, such as radiographic damage or permanent work disability. However, in order to evaluate the therapeutic response to cs-DMARD, the majority of guidelines recommend reevaluating in the short term the therapeutic response in order to adjust dosage or modifications in the therapeutic scheme. Under this concept, the results of our study are useful to describe the utility of -174G/C* IL-6* gene polymorphism to determine a subgroup of higher risk for failure in therapeutic response in patients with LEF.

We also have some strengthens in our study: we analyzed, in the same study, patients treated with LEF or MTX as monotherapy, and this aspect gave the opportunity to make comparisons between response to both drugs differently from the studies performed by Pawlik et al., who examined these groups separately [20, 21]. This strategy allowed the comparison of patients being treated with either of these two DMARDs as well as the examination of the risk that the -174G/C polymorphism of* IL-6* may confer therapeutic failure in each synthetic DMARD separately. The second and most important strength of this study is that we used a stricter definition for failure of the therapy that is according to the current concepts and goals of treatment in RA.

## 5. Conclusion

In conclusion we did not observe that the -174G/C* IL-6* gene promoter polymorphism conferred a significant risk for failure on MTX, whereas a significant risk for failure on LEF was observed after 6 months of treatment in patients carrying the GG. Future studies should evaluate whether the -174G/C* IL-6* gene promoter polymorphism can be associated with the long-term prognosis of other relevant outcome measures, such as radiographic structural damage and permanent disability in these patients. Therefore, further multicenter studies evaluating the impact of this polymorphism in a large cohort of patients with RA are required.

## Figures and Tables

**Table 1:** Comparison of selected characteristics of patients receiving methotrexate (MTX) versus leflunomide (LEF).

	MTX or LEF *n* = 96	MTX *n* = 55	LEF *n* = 41	*p*
*Sociodemographic characteristics*				
Females, *n* (%)	93 (97)	53 (96)	40 (98)	1.00
Age (yr), mean ± standard deviation	50.6 ± 10.2	48.7 ± 7.2	52.8 ± 11.0	0.06
*Disease characteristics*				
Disease duration (yr), mean ± SD	7.6 ± 7.5	6.05 ± 7.2	9.2 ± 7.6	0.05
HAQ-Di score at baseline, mean ± SD	0.95 ± 0.58	1.01 ± 0.60	0.88 ± 0.57	0.33
DAS-28 score at baseline, mean ± SD	5.6 ± 1.1	5.6 ± 1.1	5.5 ± 1.1	0.73
Rheumatoid Factor (RF) (IU/mL), mean ± SD	83.2 ± 127.4	77.4 ± 124.8	89.6 ± 132.0	0.70
Erythrocyte Sedimentation Rate (ESR) (mm/h), mean ± SD	27.1 ± 11.4	26.2 ± 12.7	28.08 ± 10.0	0.48
Interleukin-6 (IL-6) at baseline, (pg/mL) mean ± SD	19.6 ± 55.5	23.7 ± 68.7	12.9 ± 19.9	0.40
Glucocorticoid dose (mg), mean ± SD	2.0 ± 2.2	1.9 ± 1.4	2.05 ± 2.8	0.88
*Patients achieving response*				
At 3 months, *n* (%)	14 (6.8)	7 (12.7)	7 (17.1)	0.38
At 6 months, *n* (%)	12 (8.0)	7 (12.7)	5 (12.2)	0.38
*Genetic characteristics*				
Genotype GG, *n* (%)	54 (56)	33 (60)	21 (51)	0.58
Genotype GC, *n* (%)	31 (32)	17 (31)	14 (34)	
Genotype CC, *n* (%)	11 (12)	5 (9)	6 (15)	

RA: rheumatoid arthritis; IL-6: interleukin-6; MTX: methotrexate; LEF: leflunomide; HAQ-Di: Health Assessment Questionnaire-Disability index; DAS-28: modified Disease Activity Score (28 joints).

Qualitative variables were expressed in frequencies (%); quantitative variables were expressed in means ± standard deviations (SD). Comparisons between differences in proportions were performed with the chi-square test (or Fisher exact test if applicable). Comparisons between differences in means were performed with independent samples Student's *t-*tests. ^*∗*^ *p* values were obtained comparing MTX versus LEF. Response was defined as the patient achieving, at 3 or at 6 months, low disease activity or remission (DAS28, <3.2).

**Table 2:** Comparison of clinical and laboratory characteristics at the baseline between GG genotype carriers and GC or CC genotype carriers.

	GG *n* = 54	GC or CC *n* = 42	*p*
*Sociodemographic characteristics*			
Females, *n* (%)	51 (94.4)	42 (100)	0.25
Age (yr), mean ± SD	50.5 ± 9.7	50.7 ± 10.9	0.93
*Disease characteristics*			
Disease duration (yr), mean ± SD	6.9 ± 7.4	8.5 ± 7.1	0.36
DAS-28 score, mean ± SD	5.6 ± 1.1	5.5 ± 1.04	0.87
HAQ-Di score, mean ± SD	0.95 ± 0.6	0.94 ± 0.51	0.98
Rheumatoid Factor (RF) (IU/mL), mean ± SD	108.6 ± 151.9	39.1 ± 39.7	0.009
Erythrocyte Sedimentation Rate (ESR) (mm/h), mean ± SD	29.5 ± 12.3	23.8 ± 9.3	0.02
IL-6 serum levels (pg/mL), mean ± SD	25.7 ± 68.0	8.5 ± 11.8	0.08
*Treatment characteristics*			
Glucocorticoid dose (mg), mean ± SD	2.3 ± 2.7	1.5 ± 1.0	0.06
Methotrexate (MTX) *n* (%)	33 (61.1)	17 (55)	0.39
Leflunomide (LEF), *n* (%)	21 (39.0)	14 (45)	

GG: excisable homozygote genotype; GC: heterozygote genotype; CC: homozygote genotype; RA: rheumatoid arthritis; HAQ-Di: Health Assessment Questionnaire-Disability index; DAS-28: modified Disease Activity Score (28 joints); IL-6: interleukin-6 serum levels. Qualitative variables were expressed in frequencies (%); quantitative variables were expressed in means ± standard deviations (SD). Comparisons between differences in proportions were performed with chi-square test (or Fisher exact test if applicable). Comparisons between differences in means were performed using independent samples Student's *t*-tests.

**Table 3:** Evaluation of -174G/C *IL-6 *as predictor of therapeutic response to any treatment (MTX or LEF) defining nonresponse, as DAS-28 > 3.2 at 3 or 6 months in rheumatoid arthritis (RA).

Treatment with any treatment MTX or LEF, *n* = 96	Follow-up at 3 months				
	MTX and LEF nonresponse *n* = 82	MTX and LEF response *n* = 14	RR	95% CI	*p*
*Genotype*					
GG *n* = 54 (%)	47 (87.0)	7 (13.0)	—	—	0.62
GC *n* = 31 (%)	25 (80.6)	6 (19.4)	—	—	
CC *n* = 11 (%)	10 (91)	1 (9)	—	—	
GG versus GC (as referent)	—	—	1.08	0.88 to 1.32	0.20
GG versus CC (as referent)	—	—	0.96	0.77 to 1.18	0.39
GC versus CC (as referent)	*—*	*—*	0.88	0.73 to 1.14	0.40
*Genetic models*					
Dominant model (GG versus GC + CC as referent)	—	—	1.04	0.88 to 1.24	0.41
Recessive model (GG + GC versus CC as referent)	—	—	0.93	0.76 to 1.16	0.49
*Alleles 2n = 192*	*2n = 164*	*2n = 28*			
G allele, 2*n* = 139 (%)	119 (85.6)	20 (14.4)	1.00	0.88 to 1.15	0.44
C allele, 2*n* = 53 (%)	45 (84.9)	8 (15.1)	Referent	—	

	Follow-up at 6 months				
	MTX and LEF nonresponse *n* = 84	MTX and LEF response *n* = 12	RR	95% Cl	*p*

*Genotype*					
GG *n* = 54 (%)	50 (92.6)	4 (7.4)	—	—	0.13
GC *n* = 31 (%)	24 (77.4)	7 (22.6)	—	—	
CC *n* = 11 (%)	10 (90.9)	1 (9.1)	—	—	
GG versus GC (as referent)	—	—	1.19	1.07 to 1.56	**0.03**
GG versus CC (as referent)	*—*	*—*	1.01	0.81 to 1.24	0.61
GC versus CC (as referent)	*—*	*—*	0.85	0.65 to 1.11	0.31
*Genetic models*					
Dominant model (GG versus GC + CC as referent)	—	—	1.19	0.96 to 1.35	**0.05**
Recessive model (GG + GC versus CC as referent)	—	—	0.93	0.78 to 1.17	0.58
*Alleles 2n = 192*	*2n = 168*	*2n = 24*			
G allele, *n* = 139 (%)	124 (89.2)	15 (10.8)	1.07	0.94 to 1.23	0.13
C allele, *n* = 53 (%)	44 (83.0)	9 (17.0)	Referent	—	

MTX: methotrexate; LEF: leflunomide; DAS-28: Disease Activity Score for 28 joints; GG: excisable homozygote genotype; GC: heterozygote genotype; CC: homozygote genotype. Qualitative variables were expressed in frequency (%); RR: Relative Risk; 95% CI: 95% Confidence Interval. Therapeutic failure (nonresponse) was defined if patients did not achieve remission or low disease activity (DAS-28 < 3.2).

**Table 4:** Evaluation of -174G/C *IL-6 *as predictor of therapeutic response to methotrexate (MTX) defining nonresponse, as DAS-28 > 3.2 at 3 or 6 months in rheumatoid arthritis (RA).

Treatment with methotrexate (MTX), *n* = 55	Follow-up at 3 months				
	MTX nonresponse *n* = 48	MTX response *n* = 7	RR	95% CI	*p*
*Genotype*					
GG *n* = 33 (%)	30 (90.9)	3 (9.1)	—	—	0.60
GC *n* = 17 (%)	14 (82.4)	3 (17.6)	—	—	
CC *n* = 5 (%)	4 (80)	1 (20)	—	—	
GG versus GC (as referent)	—	—	1.10	0.86 to 1.41	0.32
GG versus CC (as referent)	—	—	1.13	0.72 to 1.78	0.44
GC versus CC (as referent)	*—*	*—*	1.02	0.63 to 1.68	0.67
*Genetic models*					
Dominant model (GG versus GC + CC as referent)	—	—	1.12	0.88 to 1.41	0.25
Recessive model (GG + GC versus CC as referent)	—	—	1.10	0.70 to 1.72	0.50
*Alleles 2n = 110*	*2n = 96*	*2n = 14*			
G allele, 2*n* = 83 (%)	74 (89.2)	9 (10.8)	1.09	0.90 to 1.33	0.20
C allele, 2*n* = 27 (%)	22 (81.5)	5 (18.5)	Referent	—	

	Follow-up at 6 months				
	MTX nonresponse *n* = 48	MTX response *n* = 7	RR	95% CI	*p*

*Genotype*					
GG *n* = 33 (%)	29 (87.9)	4 (12.1)	—	—	0.87
GC *n* = 17 (%)	15 (88.2)	2 (11.8)	—	—	
CC *n* = 5 (%)	4 (80)	1 (20)	—	—	
GG versus GC (as referent)	—	—	0.99	0.80 to 1.23	0.67
GG versus CC (as referent)	*—*	*—*	1.09	0.68 to 1.73	0.52
GC versus CC (as referent)	*—*	—	1.10	0.68 to 1.76	0.55
*Genetic models *					
Dominant model (GG versus GC + CC as referent)	—	—	1.01	0.82 to 1.25	0.58
Recessive model (GG + GC versus CC as referent)	—	—	1.10	0.70 to 1.72	0.50
*Alleles, 2n = 110*	*2n = 96*	*2n = 14*			
G allele, 2*n* = 83 (%)	73 (88.0)	10 (12.0)	1.03	0.86 to 1.23	0.35
C allele, 2*n* = 27 (%)	23 (85.2)	4 (14.8)	Referent	—	

MTX: methotrexate; GG: excisable homozygote genotype; GC: heterozygote genotype; CC: homozygote genotype. Qualitative variables were expressed in frequencies (%); RR: Relative Risk; 95% CI: 95% Confidence Interval. Therapeutic failure (nonresponse) was defined if patients did not achieve remission or low disease activity (DAS-28 < 3.2).

**Table 5:** Evaluation of -174G/C *IL-6 *as predictor of therapeutic response to leflunomide (LEF) defining nonresponse, as DAS-28 > 3.2 at 3 or 6 months in rheumatoid arthritis (RA).

Treatment with leflunomide (LEF) *n* = 41	Follow-up at 3 months				
	LEF nonresponse *n* = 34	LEF response *n* = 7	RR	95% CI	*p*
*Genotype*					
GG *n* = 21 (%)	17 (81)	4 (19)	—	—	0.64
GC *n* = 14 (%)	11 (78.6)	3 (21.4)	—	—	
CC *n* = 6 (%)	6 (17.6)	0 (0)	—	—	
GG versus GC (as referent)	—	—	1.03	0.73 to 1.45	0.43
GG versus CC (as referent)	—	—	0.81	0.66 to 0.99	0.17
GC versus CC (as referent)	*—*	*—*	0.78	0.60 to 1.03	0.16
*Genetic models*					
Dominant model (GG versus GC + CC as referent)	—	—	0.95	0.72 to 1.26	0.37
Recessive model (GG + GC versus CC as referent)	—	—	0.80	0.68 to 0.94	0.15
*Alleles, 2n = 82*	*2n = 68*	*2n = 14*			
G allele, 2*n* = 56 (%)	45 (80.4)	11 (19.6)	0.91	0.75 to 1.10	0.19
C allele, 2*n* = 26 (%)	23 (88.5)	3 (11.5)	Referent	—	

	Follow-up at 6 months				
	LEF nonresponse *n* = 36	LEF response *n* = 5	RR	95% CI	*p*

*Genotype*					
GG *n* = 21 (%)	21 (100)	0 (0)	—	—	**0.006**
GC *n* = 14 (%)	9 (25)	5 (100)	—	—	
CC *n* = 6 (%)	6 (16.7)	0 (0)	—	—	
GG versus GC (as referent)	—	—	1.56	1.05 to 2.30	**0.003**
GG versus CC (as referent)	*—*	*—*	1.83	1.07 to 3.14	**0.001**
GC versus CC (as referent)	*—*	*—*	0.64	0.43 to 0.95	0.06
*Genetic models *					
Dominant model (GG versus GC + CC as referent)	—	—	1.33	1.03 to 1.72	**0.02**
Recessive model (GG + GC versus CC as referent)	—	—	0.86	0.75 to 0.98	0.43
*Alleles, 2n = 82*	*2n = 72*	*2n = 10*			
G allele, 2*n* = 56 (%)	51 (91.1)	5 (8.9)	1.13	0.92 to 1.38	0.11
C allele, 2*n* = 26 (%)	21 (81.0)	5 (19.0)	Referent	—	

LEF: leflunomide; GG: excisable homozygote genotype (*n* = 21); GC: heterozygote genotype (*n* = 14); CC: homozygote genotype (*n* = 6). Qualitative variables were expressed in frequencies (%); quantitative variables were expressed as means ± standard deviations (SD). RR: Relative Risk; 95% CI: 95% Confidence Intervals. Therapeutic failure (nonresponse) was defined if patients did not achieve remission or low disease activity (DAS-28 < 3.2).
